# Neural Correlates of Trust in Automation: Considerations and Generalizability Between Technology Domains

**DOI:** 10.3389/fnrgo.2021.731327

**Published:** 2021-09-03

**Authors:** Sarah K. Hopko, Ranjana K. Mehta

**Affiliations:** Neuroergonomics Lab, Department of Industrial and Systems Engineering, Texas A&M University, College Station, TX, United States

**Keywords:** interpersonal trust, gender difference, reliability, human-robot interaction, vehicle automation

## Abstract

Investigations into physiological or neurological correlates of trust has increased in popularity due to the need for a continuous measure of trust, including for trust-sensitive or adaptive systems, measurements of trustworthiness or pain points of technology, or for human-in-the-loop cyber intrusion detection. Understanding the limitations and generalizability of the physiological responses between technology domains is important as the usefulness and relevance of results is impacted by fundamental characteristics of the technology domains, corresponding use cases, and socially acceptable behaviors of the technologies. While investigations into the neural correlates of trust in automation has grown in popularity, there is limited understanding of the neural correlates of trust, where the vast majority of current investigations are in cyber or decision aid technologies. Thus, the relevance of these correlates as a deployable measure for other domains and the robustness of the measures to varying use cases is unknown. As such, this manuscript discusses the current-state-of-knowledge in trust perceptions, factors that influence trust, and corresponding neural correlates of trust as generalizable between domains.

## Problem Statement

Trust in automation is a rising concern in many safety-critical systems due to its influence on the utilization strategy and emergent complacency behaviors of the operators. As such, measuring trust in such complex systems is essential to improve system safety and collaborative performance. In collaborative human-automation teaming, the operators can choose when and how to rely on or utilize automation features, highly dependent on how well calibrated their trust is. When trust is lower than the system's capabilities, operators tend to underutilize automated features, either by turning them off or rejecting the technology completely (Lee and See, 2004; Mouloua and Hancock, 2019). When trust is higher, operators tend to over utilize or misuse the assistance, such as continuing to use the assistance despite signs of unreliability, utilizing it in situations that the assistance was not designed for, or easily becoming distracted or complacent when automation has taken over. Currently, the state-of-the-art method to capture trust states is through surveying or interviewing the operators (Lewis et al., 2018; Hopko et al., 2021b), which, due to the discrete nature of surveys, cannot be used as a continuous real-time measure for adaptive automation or trust-sensitive detection systems. Moreover, these subjective measures are invasive and can be biased based on how the surveys are presented to a population; a worker, pilot, or driver can be biased to answer safety-related surveys following societal and employer expectations assuming they are able to stop work to answer the survey. In a research setting, surveys may also prime the participants to focus on trust, which may disrupt experimental manipulations. Due to these limitations, surveys alone cannot be readily applied to develop adaptive automation systems that can monitor and respond to trust levels real-time, nor used to mechanistically understand trust influencers' impact on perceptions and corresponding behaviors. Deployable continuous techniques, such as functional brain imaging, to objectively quantify trust in automation have promise in filling the need for an accurate and deployable measure of trust. This paper discusses the basis of neural activity as a corollary measure of trust and the potential risks of generalization between interpersonal and automation domains in addition to generalization between popular safety-critical domains such as aviation, vehicle automation, human-robot collaboration, cognitive decision aids, and automation in medical devices (Hopko et al., 2021a). This manuscript discusses the differences between interpersonal and technological domains for how trust is defined, the basis for trust perceptions, the factors that influence trust (including dispositional and gender difference), the neurological basis of trust through a review of trust-manipulated studies, and identifies the current gaps in knowledge.

## Trust Definitions in Automation

Operator trust is a complex and dynamic human factor that is impacted by a myriad of cultural, environmental, and system factor influences (Hancock et al., 2011; Schaefer et al., 2014; Chiou and Lee, 2021). Because trust is complex and dynamic in its nature, it is difficult to comprehensively operationalize the definitions of trust, regardless of technology domain. The most commonly referenced definition in technology is by Lee and See (2004) that depends on the uncertainty and vulnerability of the operator given the automated agent's actions (Chiou and Lee, 2021). While Lee and See's definition is general, some definitions of trust have domain specific divisions. For example, Hald et al. (2019) defined trust as “the combination of feeling physically safe around [the robot] and being able to predict the robot's action in context of the shared task.” While this is related to the vulnerability (safety) and uncertainty (predictability), it is a more specific application of Lee and See's definition as vulnerability is specified to physical safety, excluding potential fiscal loss, workload changes, or connected affect state. Similar specification of trust definitions has been observed in other safety critical domains, including cognitive aids and alarms that focus more on system reliability than safety (Madhavan et al., 2006; Parasuraman et al., 2008). While definitions are similar, the traditional survey measurements are influenced by the nuances in definitions. A literature survey by Lewis et al. (2018) found that the majority of utilized trust surveys are un validated, i.e., they are developed study-specific by the researchers. Given specializations of trust definitions across domains, and that many surveys are developed based on these definitions, there are nuanced differences between technology domains on what trust is, the importance of factors that influence it, and questions that are relevant to trust perceptions. In analyzing two validated trust surveys, one generic to trust in automation and another specific to trust in robotics, Kessler (2020) found that the surveys were not interchangeable and were capturing distinct trust perceptions. Given that many studies that investigate neural correlates of trust validate their response to subjective questionnaires, there is a need for the trust domain to unify trust definitions, trust models, and measurement techniques (i.e., surveys, physiological responses), or to acknowledge domain specific findings.

## Basis of Trust in Interpersonal Verses Technology Automation

Comparison of trust between interpersonal collaborative domains and technologies is rare, especially with regard to the generalizability of neural signatures (Parasuraman et al., 2014). Madhavan and Wiegmann (2007) performed a literature review illustrating that the predominate bases of trust differ between interpersonal trust and human-technology trust. Interpersonal trust is based on three major rationales: (1) the integrity of the trustee (i.e., how lawful and of good moral), (2) the ability of the trustee (i.e., their capabilities to accomplish the desired interaction/task), and (3) the benevolence of the trustee (i.e., how Good, or altruistic, an individual is). Another investigation notes that interpersonal trust is also influenced by the familiarity between the trusting parties, shared experiences, shared goals, reciprocal discloser, and demonstration of non-exploitation, all expressed over long-durations (Dani et al., 2006). Interpersonal trust directly differs from trust in technology in two ways. The first is that technology lacks intentionality, unlike humans. Automation is based on scripts, system capabilities, and algorithms scoped to a specific use case. Thus, automation cannot truly develop its own intents unlike humans (Madhavan and Wiegmann, 2007; Charalambous et al., 2016), although its intents may be reflective of the designer's (i.e., human) biases. Moreover, the use of automation in safety critical systems are assumed to be designed such that it improves the system or a subcomponent of the system; users may assume automation is intended to work in support of them. Therefore, the reciprocal discloser, demonstration of non-exploitation, or other anthropomorphic traits tend to be less relevant than they are in interpersonal trust. As such, the perceived capability of the automated system has been deemed as the primary basis for trust in automation (Chen et al., 2018).

The second major difference between interpersonal trust and trust in automation is the lack of anthropomorphism (in many technologies) and accompanying societal expectations. While benevolence and integrity are not directly attributable to the technology, users are able to personify technology (Nass and Moon, 2000). Systems that parallel human-like characteristics and personas (i.e., humanoid robots, intelligent agents such as Alexa) tend to have more trust than systems designed with the same capacities and purpose, but with non-anthropomorphized characteristics (Hancock et al., 2011; de Visser et al., 2017; Calhoun et al., 2019). However, there is a point where extreme similarity between a technology and human can result in a significant drop in trust levels, often referred to as the uncanny valley (Flemisch et al., 2017). Because of this interaction between trust and human-like characteristics in technology, there are observable differences in trusting behavior, founded on emotional connection to the system rather than system capability (Jensen et al., 2020). Given the spatial association of cognitive and emotional systems in the brain, it is conceivable that these trust differences are observable in neural activity between interpersonal, anthropomorphized technology, and non-anthropomorphized technology trust.

## Factors Influencing Trust in automation

Trust is influenced by human factors, automation factors, and environmental factors (de Visser and Parasuraman, 2011; Hancock et al., 2011; Schaefer et al., 2014; Lewis et al., 2018). Example human factors include demographics and user characteristics (age, gender, culture, race, ethnicity, personality, etc.), situational factors (mood, fatigue, affect, vigilance, task engagement, etc.), and user attributes (mental workload capacity, capability, expertise, etc.). Example automation factors include the purpose of the system, the process in which the system completes its task, automation level, the system attributes (size, safety features, etc.), in addition to the capability, accuracy, reliability, or ease of use of the system (Hoff and Bashir, 2015). Environment factors depend on the scoped boundary of the system, but can include interaction with other systems, physical layout and proximity to the system, cultural, and societal factors, weather and others (Hancock et al., 2011). The following review will compare and contrast these factors in the broad sense of safety critical automation domains. Because the use cases, and thus goals, tasks, strategies, and perceptions, are different, we posit that the tendencies of trust perceptions differ between technologies, potentially changing the accompanying neural signatures.

### Perceptual Basis of Trust

While the magnitude and variance may change between domains, trust is comprised of three, highly interrelated components: dispositional, situational, and learned (Hancock et al., 2011). Dispositional trust is the reasonably static underlying tendency to trust the automation in general, and can be influenced by several factors, such as demographics, culture, race, age, gender, etc. Situational trust is the trusting behavior based on both internal factors (i.e., mood, engagement, fatigue), and external situational factors (i.e., environment, system process, features). Learned trust is a dynamic mental model of the trustworthiness of a system as one gains familiarity, both through the reputation of the system and first-hand interaction. While these components are not completely separable, studies primarily focus on manipulating situational instances, such as reliability and environmental factors, or manipulate situational-learned components such as the influence of reliability (or unreliability) over time or user experience with a specific task. The neural activity associated with these manipulations relies heavily on cognitive ability to determine an associated mental model of the reliability and risk, such as Bayesian mental models (Adolphs, 2002). It is clear that emotional or affect factors also influence trust perceptions as anxiety, hostility, and negative attitudes are often measured alongside trust perceptions (Hopko et al., 2021b), although emotional studies of trust are scarce (Jensen et al., 2020).

#### Dispositional and Gender Differences in Trust Perceptions

The impact of dispositional trust on overall trust perceptions has been commonly overlooked, where most trust-manipulation studies focus on event-based trust breaches or system wide changes that manipulate trust (Parasuraman et al., 2008). Dispositional trust is key as the generalization of the importance of trust influencers (e.g., reliability, cyber security, accuracy) requires understanding of how users value trust influencers in individual domains. This is needed in order to build robust designs given the user population and predisposition to utilize the technology. For example, gender differences have been observed in both technology (Syrdal et al., 2007; Kuo et al., 2009; Strait et al., 2015) and interpersonal trusting behavior (Croson and Buchan, 1999). Furthermore, there have been gender differences in societal values, where women have historically been more implicated in healthcare and the well fair of the family than men, making ~80% of decisions for their families (Matoff-Stepp et al., 2014). This has resulted in healthcare marketing strategies that target women more often than men. However, other domains, such as vehicle automation and industrial robotics, do not have similar marketing or training strategies, which may have implications for both safety and technology-focused workforce development. Because gender is a factor in trusting behavior, and gender has domain-specific characteristics and values, there may be rationale for dispositional trust differences between these technologies. This can be observed in a pilot survey conducted by the authors (Hopko et al., 2021a), where the traditional measure for dispositional trust, using a propensity to trust automation questionnaire (Jessup et al., 2019), was found to correlate with trust more strongly in cyber/cognitive aid technologies than any other investigated domain (e.g., medical, robots, vehicle).

#### Situational and Learned Differences in Trust Perceptions

The underlying trust behaviors are also likely to be different due to the familiarity and experience with a domain. The average knowledge of automation, given cultural standards, varies between applications; people are more familiar with vehicle automation than they are of collaborative robotics (Hopko et al., 2021a) likely because it is a more commercialized technology; moreover, aviation automation is a normality for pilots (Trösterer et al., 2017). Furthermore, as these domains have different working environments and different tasks, the severity of situational trust factors may not be directly generalizable between domains; a reliability of 90% may be acceptable for diagnostic systems but unacceptable for vehicle automation (Wickens and Dixon, 2007). Because each technology domain is a different use case, the relevance of trust influencers (e.g., reliability) to operators and the socially acceptable behaviors (e.g., reliability thresholds) of the systems differ between domains. The importance of traditional trust scales, namely reliability, accuracy, or ease of use, have been shown to differ between technology domains, such as accuracy being most important in medical device automation, and reliability most important in vehicle and robots (Hopko et al., 2021a). Thus, while there is overlap in many of these technologies (e.g., vehicles can have cyber aid alarm systems), the magnitude and relevance of trust influencers, or trusting behaviors, between domains need independent considerations.

The three trust components of trust and their interdependency is likely to differ between domains. The extent to which these differences would influence physiological correlates is currently unknown. It is likely that physiological responses, such as dermal activity, heart rate activity, etc., lack the spatial resolution offered by neurological responses to capture these distinctions. For example, an increase in heart rate may signal increased physical workload (Roscoe, 1992; Garet et al., 2005), but also an anxious response (Perrotta, 2019), while distinct changes in the amygdala, the hippocampus and the prefrontal cortex may signal specific onset of anxiety (Perrotta, 2019). The initial biases of participants toward trusting certain technologies more than others and the grounds for which trust perceptions are founded hint that accompanying neural and physiological responses may differ. The magnitude of the neurological responses, and brain function-specific activation patterns, may also differ between domain due to these attitudes and emotional connection to certain technologies, which has been shown to influence neural activity (Larsen et al., 2008).

## Neural Basis of trust

### Introduction to Popular Brain Imaging Techniques

There is no better place to capture a subjective human state than at the source: the brain. The non-invasive brain imaging techniques discussed in this section are measurable corollary responses of trust. Functional magnetic resonance imaging (fMRI) is considered the gold-standard for mechanistic brain imaging due to its increased spatial resolutions; however, it has reduced temporal resolution, high cost, and is not ambulatory (Logothetis, 2008; Table 1). In contrast to fMRI, electroencephalogram (EEG) and functional near-infrared spectroscopy (fNIRS) are ambulatory measures; they can measure brain activity in real-time during a task with higher temporal resolution, although are limited to measuring cortical brain activity with lower spatial resolution (Ferrari and Quaresima, 2012; Mehta and Parasuraman, 2013).

**Table 1:** Summary of popular brain imaging techniques.

**Imaging Method**	**fMRI**	**fNIRS**	**EEG**
Spatial Ranking	High	Mid-High	Mid
Temporal Ranking	Low	Mid	High
Cost	Expensive	Affordable	Upper-Affordable
Measurement	Hemoglobin Dynamics	Hemoglobin Dynamics	Electrical Activity
Ambulatory		✓	✓
Measurable Locations	Cortical + Subsurface	Cortical	Cortical

### Review of Neural Correlates of Trust in Automation

Here we review studies that manipulate trust in automation and measure accompanying neural correlates. Studies were identified using the following three search term groups in the title, keywords, or abstract of the paper (Table 2) in addition to reviewing references of included papers. A total of eleven studies were included based on the criteria of (1) the study focused on trust in any type of automation, (2) the study must have manipulated trust and measured accompanying neural activity, (3) the study must have reported the locations that correlated with trust condition. Studies that applied machine learning techniques to all measured brain locations but did not provide which locations were significant contributors were excluded. And, (4) the studies were not focused on highly anthropomorphized technology. While anthropomorphism was allowed in the included studies, highly anthropomorphized technology (e.g., gendered humanoid robots, systems with emotions) were excluded from this synthesis because high anthropomorphism influences the bases for which trust is provided as discussed in section Basis of trust in interpersonal verses technology automation.

**Table 2:** Search terms.

**Group**	**Terms**
Brain	neural correlates, neural signatures, neural patterns, neural features, brain, neuro, fMRI, EEG, fNIRS
Trust	trust, distrust, overtrust, undertrust, mistrust
Technology	robot, automation, technology, tech, machine, artificial intelligence, computer, vehicle, aviation, pilot, air traffic, operator

**Figure 1:**
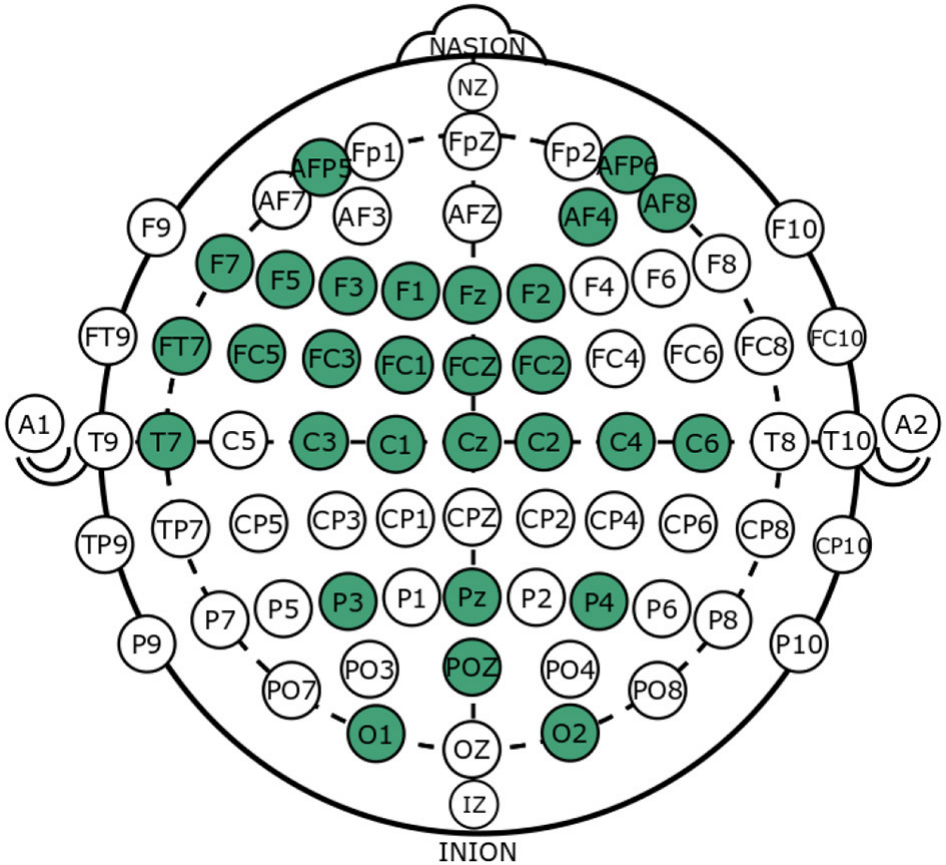
Binary summary of neural correlates of trust in technology domain. All green locations have bee shown to vary by trust condition in at least one study (refer to Table 3).

The locations that were found to correlate with trust are defined based on Brodmann area or, for cortical locations, the 10–20 international standard, whose locations are universally denoted as follows: Nasion (N), Frontal (F), Central (C), Parietal (P), Occipital (O), and Inion (I). Table 3 summarizes studies' trust manipulation methods, imaging method, and locations that were significantly different between trust-manipulated conditions. Figure 1 is the visualization of the identified locations, where each green location was found to correlate with trust in at least one study. The primary method to capture neural activity was with EEG, where the remaining papers utilized fMRI. The majority of the studies included looked into artificial intelligence or computer algorithms with correct and incorrect responses at different reliability rates (Dong et al., 2015; Goodyear et al., 2016; Akash et al., 2018; de Visser et al., 2018; Wang et al., 2018; Ajenaghughrure et al., 2019; Eun-Soo et al., 2019; Pushparaj et al., 2019; Sanders et al., 2019). It was shown that humans react differently to correct and incorrect actions of the agent, and these reactions correlate with subjective trust scores, with accompanying neural activation.

**Table 3:** Summary of neural correlates of trust in technology domains.

**Paper**	**Trust manipulation method**	**Imaging method**	**Locations correlated with trust manipulation**
Wang et al. (2018)	Autonomous agents are designated as “Trustworthy” or “Untrustworthy” each with different probabilities of getting a financial return.	EEG	AFP5, F7, F5, F3, AF7, AF5, AF3, F1, Fz, C1, Cz, AFP6, AF6, AF8, POz, O1, O2
Eun-Soo et al. (2019)	Human and robot faced agents with different risk-taking natures who provide advice with a set reliability rate.	EEG	Cz, FC1, FC2
Ajenaghughrure et al. (2019)	*Who Wants to be a Millionaire* game with different question difficulties and points per question with human or computer help line	EEG	C3, C4, P4, POz
Hu et al. (2016)	Vehicle obstacle detection with reliability of 100% or 50% that considers trust over time.	EEG	Cz, C4
de Visser et al. (2018)	Agent has credibility (expert, novice) with correct response reliability rate (90, 60)%	EEG	region centered at FCz
Dong et al. (2015)	Prisoner's dilemma type game against human or computer agent with collaborative or egoism strategies.	EEG	Fz, Cz, Pz, FC1, FC2
Goodyear et al. (2016, 2017)	X-ray luggage-screening task with human or computer agent advice each with 60% reliability at detecting a knife.	fMRI	right rostrolateral-PFC, right V1, right and left pre-SMA, right orbito-FC
Sanders et al. (2019)	Two levels each of reliability and credibility	EEG	Anterior cingulate cortex, centered at FCz
Pushparaj et al. (2019)	Aircraft conflict with accurate alerts at 5 levels of detection difficulty and shown videos of potential conflicts.	fMRI	Insular cortex, amygdala, putamen, nucleus accumbens, anterior and posterior cingulate cortex
Akash et al. (2018)	Obstacle detection alert with reliability of 100% or 50% where the operator must choose to take or ignore advice	EEG	POz, P3, Fz, C3, C4, Cz

#### Activation Features

Neural activation is the primary feature extracted from neural activity. It illustrates the strength to which a region, or location, is responding to a stimulus. The study conducted by de Visser et al. (2018) predicted that a mismatch between expected and actual outcomes in artificial intelligence agents would result in a growing negative potential in the anterior cingulate cortex, which can be measured in the front ocentral scalp region centered around FCz. The authors confirmed that correct vs. error responses have different neural signatures and that different levels of reliability, but not credibility, impact the magnitude of the activation; stronger activation occurs in the intermediate frontal cortex, supplementary motor area, and premotor cortex when there is an error in highly reliable situations. They also report that this activation negatively correlates with human trust; an increase in activation suggests lower trust. Validation of this experiment was perfomed by Sanders et al. (2019), who also observed increased activation of the region around FCz during unreliable conditions. All EEG studies that manipulated reliability report similar findings, where at least one of the locations Fz, F1, F2, FC1, FCz, FC2, C1, Cz, C2 are identified with identical activation direction (increased activity for lower trust). The fMRI study (Goodyear et al., 2016, 2017) also identified the left and right pre supplementary motor area, located around FCz, which overlaps with the regions identified in the aforementioned EEG-based trust studies. Time-domain features were predominately found significant for EEG features in the frontocentral region locations that are centered around FCz (Akash et al., 2018; Eun-Soo et al., 2019), similar to that identified in a literature review of EEG brain-computer-interface features (Lotte et al., 2007).

For studies that did not manipulate reliability, there was more variance in the locations that correlate with trust conditions. For example, in an investigation that had agents provide recommendations for a financial investment with three different underlying probabilities of return, the locations that were found to vary by probability were primarily located in the left, medial, and right frontal lobe and occipital lobe (Wang et al., 2018). The investigations into game theory strategies and game show tasks also identified the locations in the parental cortex and occipital cortex (Dong et al., 2015; Ajenaghughrure et al., 2019). While these investigations focus on cyber aid technology, the slight differences in the studies as they move farther away from detection systems (namely, game theory studies) illustrates the need to consider the generalizability of these correlates to different use cases and trust influencers. These correlates may only identify the influence of reliability to trust perceptions, rather than robust to any trust influencer, such as gender.

Four of the studies also compared human or anthropomorphized agents to non-anthropomorphized agents (Table 4), all of which observed differences in trusting behaviors and neural correlates (Dong et al., 2015; Goodyear et al., 2016, 2017; Eun-Soo et al., 2019). In addition to distinct neural patterns between the reliable and unreliable conditions in the intermediate frontal cortex, Eun-Soo et al. (2019) reported that the strength of activation for unreliable conditions were more noticeable for human-faced assistive agents than for robot faced agents. Similarly, Goodyear observed higher activation for unreliable human-agents in the insular cortex, somatosensory association cortex/ visuo-motor coordination, agranular retrolimbic area, anterior prefrontal cortex, and superior temporal gyrus during the first trials of the task as compared to machine-agents. When participants observed feedback on their decision, increased activation for human-agents who provided good advice was observed in the left dorsomedial prefrontal cortex and the medial frontal gyrus (BA 9/10). Furthermore, their participants were more likely to follow the advice of a human-agent and perceived the machine-agent advice as more unreliable even though the objective reliability and subjective trust scores were similar between the two groups.

**Table 4:** Summary of activation features for trust-agent type interaction.

**Paper**	**Agent types investigated**	**Imaging methods**	**Trust-agent type interaction**
Dong et al. (2015)	Human verses computer agent	EEG	Increased mean amplitudes ERPs is associated with human-like cues. And, increased visual saliency affects strongly correlated with perceived capability of the teammate.
Eun-Soo et al. (2019)	Human verses robot faced agents	EEG	Strength of activation for theta frequency band in unreliable conditions are more noticeable for human-faced agents.
Goodyear et al. (2016, 2017)	Human verses machine agent	fMRI	Higher activation early on for human agents in the insular cortex, somatosensory association cortex, agranular retrolimbic area, anterior PFC, superior temporal gyrus. When receiving feedback, Increased activation for human agents who provided good feedback in the left dorsomedial PFC and medial frontal gyrus.

Dong et al. identified varying event-related potentials such as increased mean amplitudes associated with human-like cues and increased visual saliency affects that strongly correlated with the participants' perceived capability of the teammate. Similar cues were not identified in the conditions without human-like cues. These findings demonstrate the physical and behavioral anthropomorphism of a machine teammate impacts trust perceptions, where increased human-like features result in increased trust, measurable in neural correlates.

#### Connectivity Features

In addition to activation features, connectivity features can provide context into which regions are functionally working together or driving activations in other regions. Two main types of connectivity analysis are traditionally employed: functional connectivity and effective connectivity. Functional connectivity is the non-directional coupling of regions whereas effective connectivity is a directional coupling. Only three of the studies performed any type of connectivity analysis (Table 5). In addition to identifying significant locations, Goodyear et al. (2016, 2017) also used granger causality, a type of effective connectivity analysis. When comparing human and machine agents, they found that the left anterior precuneus and the posterior insula are drivers of the trust network with influences on all other significant regions of interest identified as correlates after FDR corrections (namely, right anterior precuneus, left posterior temporoparietal junction, posterior cingulate cortex, and left rostro lateral prefrontal cortex). They argue that the left anterior pre-cuneus and posterior insula jointly work together by integrating social and logical evaluations with internal interception responses. Granger causality was also used by Sanders et al. (2019), although no specific conclusions are reported due to the limited sample size of the study. They do suggest that the anterior and posterior cingulate cortex may be important nodes to consider in connectivity networks, and that the total flow and asymmetry of the network may be important features. The last study used seed-based connectivity, a functional connectivity analysis (Pushparaj et al., 2019). For all five levels of task difficulty, the anterior cingulate cortex was a strong network seed connected to the insular cortex, and in more difficult tasks, the putamen. The insular cortex was similarly a strong seed in the functional connectivity network connected to the anterior cingulate cortex, insular cortex network, putamen, and nucleus accumbens.

**Table 5:** Summary of connectivity features.

**Paper**	**Connectivity type**	**Imaging methods**	**Connectivity features correlating with trust**
Sanders et al. (2019)	Effective	EEG	No specific conclusions due to limited sample size, although they do posit the anterior and posterior cingulate cortex
Goodyear et al. (2016, 2017)	Effective	fMRI	The left anterior precuneus and posterior insular are drivers of the trust network with influences on all other significant activation regions
Pushparaj et al. (2019)	Functional	fMRI	Anterior cingulate cortex and insular cortex are strong seeds of the network, highly connected to other regions including the putamen and nucleus accumbens

### Considerations for Neural Correlates of Human-Automation Trust

There is consensus within the current studies that trust in automation can be monitored using neural signatures, frequently identified in the fronto-central region, including areas functionally named as the intermediate frontal cortex, primary motor cortex (MC), pre-MC, and supplementary motor area (SMA), potentially influenced by the anterior or posterior cingulate cortex. It is not surprising that the primary MC, pre-MC, and SMA were recognized in technology agent studies, as they are responsible for planning and executing motor actions based on internal and external cues. These areas also have overlap with the intermediate frontal cortex that is thought to be responsible for managing uncertainty (Ferng, 2020). These locations could potentially provide a continuous measurement of trust to be used for trust calibration, in addition to providing a mechanistic understanding of trust influencers.

While the prefrontal cortex (PFC) was not found significant in most of the papers, our review identified its relevance. It has been previously observed that trust and workload act a co-varying entities, where an increase in workload results in a decrease in trust (Chen et al., 2011; Hu et al., 2016). As the PFC, responsible for complex cognition and working/short-term memory, was identified by a subset of the reviewed articles, investigations into the neural correlates of trust should control for the co-varying influence of workload on trust perceptions in the accompanying neural signatures. Within interpersonal trust, there is a similar consensus that the cognitive system of trust is primarily comprised of the ventrolateral prefrontal cortex and amygdala and that trust is influenced by deeper brain regions linked to the motivational (risk-reward) and risk cognition systems and to social affect systems (Adolphs, 2003; Yanagisawa et al., 2011). Trust in automation has primarily been discussed similar to risk cognition, where trust is characterized by user uncertainty and vulnerability (Lee and See, 2004), and risk cognition itself has often been measured alongside trust or as subscales of trust (Hopko et al., 2021b). Thus, one can posit that trust relies on ability or willingness to perceive risk in addition to the willingness to be subject one's self to the consequence. There are three identified candidates responsible for the risk-reward evaluation of decision alternatives: the amygdala, ventral striatum, and orbitofrontal cortex (Drnec et al., 2016). The importance of these regions lies on the cognition of an action; the amygdala and lateral orbitofrontal cortex are thought to be responsible for interpreting the negative risk of an action, whereas the ventral striatum and medial orbitofrontal cortex are responsible for the perceiving the rewards (Basten et al., 2010).

## Gaps in Neural Correlates Of Trust Literature

Most of the summarized studies in Table 3 are exploratory or pilot studies that compared neural correlates of human verses technology agents, as the neural correlates of trust have been predominantly studied in interpersonal and reciprocal trust (Parasuraman et al., 2014). These studies reported small sample sizes in highly controlled environments. Many of these studies that compared human with technology agents found statistically significant differences in brain activity between agent type, supporting the claim that people trust technology agents differently than they trust humans (Madhavan and Wiegmann, 2007), measurable in neural activity. There is limited work on neural correlates of trust in automation, where the vast majority of current studies are in cyber aid/detection system technologies. It is still unknown how well the neural correlates of trust will generalize between automation domains.

There is a lot left to unravel about what features in the brain signal trust levels and whether brain activity can capture changes in trust perceptions (i.e., trust building, breach, and/or repair). Systematic investigation into these time-dependent trust markers is warranted to understand, measure, and model effective human-automation trust calibration. Furthermore, mis-calibrated levels of trust do not always influence the user's behavior (Chiou and Lee, 2021). As such, the neural correlates associated with a user's identification of a trust influencer and the decision to act upon the trust perception are needed. The studies reviewed here highlight the potential of using neural signatures, both through activation and connectivity features, in better capturing operator trust in automation. However, only three studies performed any form of connectivity analysis. When data mining the neural correlates of trust, it is important to consider not only which regions are effectively responsible for driving the activations, but also how the different regions of interest are functionally communicating with each other. Connectivity analysis can be used to better understand trust adaptations in different conditions rather than just a snapshot of activations. Furthermore, the predominant method to capture the neural correlates of trust in existing literature is EEG, which has a disadvantage when measuring region specific dynamics due to poor spatial resolutions. Other neural imaging methods, like fNIRS, have an advantage of offering higher spatial resolution, are less invasive, and are cost effective, and thus might provide additional information, perhaps implemented alongside or independently of EEG.

## Concluding Remarks

This review aimed to provide context onto the relevance of trust-related findings in interpersonal and automation domains in addition to context about the generalizability of trust between various automation domains. Due to the novelty of the neural correlates of trust in technology, there is a dearth of works outside cyber aid technologies. There is still need to understanding trust in other automation domains beyond cyber aid technologies and beyond trust influencers like “reliability” such as investigating how the impacts of “new age” trust influencers [i.e., team autonomy and fluency; (Chiou and Lee, 2021)] affect trust perceptions and corresponding neural correlates. For the studies that consider agent type (namely, human or anthropomorphic agents against technology agents), there were observable differences in the neural correlates of trust. Future research is warred to investigate the similarities or differences between automation levels and interpersonal relation levels as pertaining to trust and its corresponding neural activity. The use of neural activity can provide unique insights other bioinstrumentations may be unable to capture due to its spatial resolution and location-based functionality. Beyond the use of traditional subjective measures, objective correlates of trust can provide high resolution into the temporal aspects of trust and provide a real-time measure for use in trust-sensitive technology. In doing so, automation can be sensitive to physiological indicators of trust and respond accordingly. In short, a neuro ergonomics approach may prove promising to better understand and model human-automation trust.

## Author Contributions

SH and RM contributed to the conception of the study. SH performed the review and drafted the first version of the manuscript. SH and RM wrote sections of the manuscript. All authors contributed to the manuscript revision, read, and approved the submitted version.

## Conflict of Interest

The authors declare that the research was conducted in the absence of any commercial or financial relationships that could be construed as a potential conflict of interest.

## Publisher's Note

All claims expressed in this article are solely those of the authors and do not necessarily represent those of their affiliated organizations, or those of the publisher, the editors and the reviewers. Any product that may be evaluated in this article, or claim that may be made by its manufacturer, is not guaranteed or endorsed by the publisher.

## References

[B1] Adolphs R. (2002). Trust in the brain. Nat. Neurosci. 5, 192–193. 10.1038/nn0302-192 11865307

[B2] Adolphs R. (2003). Cognitive neuroscience: cognitive neuroscience of human social behaviour. Nat. Rev. Neurosci. 4, 165–178. 10.1038/nrn1056 12612630

[B3] Ajenaghughrure I. Ben Sousa S. C. Kosunen I. J. Lamas D. (2019). Predictive model to assess user trust: a psycho-physiological approach. Proc. 10th Indian Conf. Hum. Comput. Interact. 1–10. 10.1145/3364183.3364195

[B4] Akash K. Hu W.-L. Jain N. Reid T. (2018). A classification model for sensing human trust in machines using EEG and GSR. ACM Trans. Interact. Intell. Syst. 8, 1–27. 10.1145/3132743

[B5] Basten U. Biele G. Heekeren H. R. Fiebach C. J. (2010). How the brain integrates costs and benefits during decision making. Proc. Nat. Acad. Sci. 107, 21767–21772. 10.1073/pnas.0908104107 21118983 PMC3003102

[B6] Calhoun C. S. Bobko P. Gallimore J. J. Lyons J. B. (2019). Linking precursors of interpersonal trust to human-automation trust: an expanded typology and exploratory experiment. J. Trust Res. 9, 28–46. 10.1080/21515581.2019.1579730

[B7] Charalambous G. Fletcher S. Webb P. (2016). The development of a scale to evaluate trust in industrial human-robot collaboration. Int. J. Soc. Robot. 8, 193–209. 10.1007/s12369-015-0333-8

[B8] Chen J. Y. C. Barnes M. J. Kenny C. (2011). Effects of unreliable automation and individual differences on supervisory control of multiple ground robots. 2011 6th ACM/IEEE Int. Conf. Hum. Robot Interact. 371–378. 10.1145/1957656.1957793

[B9] Chen M. Nikolaidis S. Soh H. Hsu D. Srinivasa S. (2018). Planning with trust for human-robot collaboration. Proceed. 2018 ACM/IEEE Int. Conf. Hum. Robot Interact. 307–315. 10.1145/3171221.3171264

[B10] Chiou E. K. Lee J. D. (2021). Trusting automation: designing for responsivity and resilience. Hum. Factors 00187208211009995. 10.1177/00187208211009995 33906505

[B11] Croson R. Buchan N. (1999). Gender and culture: international experimental evidence from trust games. Am. Econ. Rev. 89, 386–391. 10.1257/aer.89.2.386

[B12] Dani S. S. Burns N. D. Backhouse C. J. Kochhar A. K. (2006). The Implications of organizational culture and trust in the working of virtual teams. Proceed. Instit. Mech. Eng. Part B: J. Eng. Manufact. 220, 951–960. 10.1243/09544054JEM415

[B13] de Visser E. Parasuraman R. (2011). Adaptive aiding of human-robot teaming: effects of imperfect automation on performance, trust, and workload. J. Cognit. Eng. Decis. Mak. 5, 209–231. 10.1177/1555343411410160

[B14] de Visser E. J. Beatty P. J. Estepp J. R. Kohn S. Abubshait A. Fedota J. R.. (2018). Learning from the slips of others: neural correlates of trust in automated agents. Front. Hum. Neurosci. 12:309. 10.3389/fnhum.2018.00309 30147648 PMC6095965

[B15] de Visser E. J. Monfort S. S. Goodyear K. Lu L. O'Hara M. Lee M. R.. (2017). A little anthropomorphism goes a long way: effects of oxytocin on trust, compliance, and team performance with automated agents. Hum. Factors 59, 116–133. 10.1177/0018720816687205 28146673 PMC5477060

[B16] Dong S.-Y. Kim B.-K. Lee K. Lee S.-Y. (2015). A preliminary study on human trust measurements by eeg for human-machine interactions. Proceed. 3rd Int. Confer. Hum. Agent Interact. 265–268. 10.1145/2814940.2814993

[B17] Drnec K. Marathe A. R. Lukos J. R. Metcalfe J. S. (2016). From trust in automation to decision neuroscience: applying cognitive neuroscience methods to understand and improve interaction decisions involved in human automation interaction. Front. Hum. Neurosci. 10:290. 10.3389/fnhum.2016.00290 27445741 PMC4927573

[B18] Eun-Soo J. Suh-Yeon D. Soo-Young L. (2019). Neural Correlates of Variations in Human Trust in Human-like Machines during Non-reciprocal Interactions. Sci. Rep. 9, 1–10. 10.1038/s41598-019-46098-8 31292474 PMC6620272

[B19] Ferng A. (2020). Brodmann Areas. Kenhub. Available online at: https://www.kenhub.com/en/library/anatomy/brodmann-areas

[B20] Ferrari M. Quaresima V. (2012). A brief review on the history of human functional near-infrared spectroscopy (fNIRS) development and fields of application. Neuroimage 63, 921–935. 10.1016/j.neuroimage.2012.03.049 22510258

[B21] Flemisch F. Altendorf E. Canpolat Y. Weßel G. Baltzer M. Lopez D.. (2017). Uncanny and unsafe valley of assistance and automation: first sketch and application to vehicle automation, in Advances in Ergonomic Design of Systems, Products and Processes, eds Schlick C. M. Duckwitz S. Flemisch F. Frenz M. Kuz S. Mertens A. Mütze-Niewöhner S. (New York, NY: Springer), 319–334.

[B22] Garet M. Boudet G. Montaurier C. Vermorel M. Coudert J. Chamoux A. (2005). Estimating relative physical workload using heart rate monitoring: a validation by whole-body indirect calorimetry. Eur. J. Appl. Physiol. 94, 46–53. 10.1007/s00421-004-1228-9 15609030

[B23] Goodyear K. Parasuraman R. Chernyak S. de Visser E. Madhavan P. Deshpande G.. (2017). An fMRI and effective connectivity study investigating miss errors during advice utilization from human and machine agents. Soc. Neurosci. 12, 570–581. 10.1080/17470919.2016.1205131 27409387

[B24] Goodyear K. Parasuraman R. Chernyak S. Madhavan P. Deshpande G. Krueger F. (2016). Advice taking from humans and machines: an fmri and effective connectivity study. Front. Hum. Neurosci. 10:542. 10.3389/fnhum.2016.00542 27867351 PMC5095979

[B25] Hald K. Rehm M. Moeslund T. B. (2019). Proposing human-robot trust assessment through tracking physical apprehension signals in close-proximity human-robot collaboration, in 2019 28th IEEE International Conference on Robot and Human Interactive Communication (RO-MAN), Robot and Human Interactive Communication (RO-MAN), 2019 28th IEEE International Conference On, 1–6. edseee.

[B26] Hancock P. A. Billings D. R. Schaefer K. E. Chen J. Y. C. de Visser E. J. Parasuraman R. (2011). A meta-analysis of factors affecting trust in human-robot interaction. Hum. Fact. 53, 517–527. 10.1177/0018720811417254 22046724

[B27] Hoff K. A. Bashir M. (2015). Trust in automation: integrating empirical evidence on factors that influence trust. Hum. Fact. 57, 407–434. 10.1177/0018720814547570 25875432

[B28] Hopko S. Mehta R. McDonald A. D. (2021a). Trust in automation: comparison of automobile, robot, medical, and cyber aid technologies. Proc. Hum. Fact. Ergon. Soc. Ann. Meet.

[B29] Hopko S. Wang J. Mehta R. K. (2021b). Human Factor Considerations and Metrics in Shared Space Human-Robot Collaboration. IEEE Systems. 10.3389/frobt.2022.799522 PMC8850717 35187093

[B30] Hu W. Akash K. Jain N. Reid T. (2016). Real-time sensing of trust in human-machine interactions. IFAC-PapersOnLine. 49, 48–53. 10.1016/J.IFACOL.2016.12.188

[B31] Jensen T. Khan M. M. H. Albayram Y. Fahim M. A. A. Buck R. Coman E. (2020). Anticipated emotions in initial trust evaluations of a drone system based on performance and process information. Int. J. Hum. Comput. Interact. 36, 316–325. 10.1080/10447318.2019.1642616

[B32] Jessup S. Schneider T. Alarcon G. Ryan T. Capiola A. (2019). The Measurement of the Propensity to Trust Automation. ResearchGate. Available online at: https://www.researchgate.net/publication/334344580_The_Measurement_of_the_Propensity_to_Trust_Automation

[B33] Kessler T. (2020). Neurophysiological Correlates of Trust in Robots. Electronic Theses and Dissertations. Available online at: https://stars.library.ucf.edu/etd2020/239

[B34] Kuo I. H. Rabindran J. M. Broadbent E. Lee Y. I. Kerse N. Stafford R. M. Q.. (2009). Age and gender factors in user acceptance of healthcare robots. RO-MAN 2009-−18th IEEE Int. Sympos. Robot Hum. Interact. Commun. 532, 214–219. 10.1109/ROMAN.2009.5326292

[B35] Larsen J. T. Berntson G. G. Poehlmann K. M. Ito T. A. Cacioppo J. T. (2008). The psychophysiology of emotion, in Handbook of Emotions, 3rd ed (New York, NY: The Guilford Press), 180–195.

[B36] Lee J. D. See K. A. (2004). Trust in automation: designing for appropriate reliance: Hum. Fact. 46, 50–80. 10.1518/hfes.46.1.50.30392 15151155

[B37] Lewis M. Sycara K. Walker P. (2018). The role of trust in human-robot interaction, in Foundations of Trusted Autonomy, eds Abbass H. A. Scholz J. S Reid D. J. (New York, NY: Springer International Publishing), 135–159.

[B38] Logothetis N. K. (2008). What we can do and what we cannot do with fMRI. Nature 453, 869–878. 10.1038/nature06976 18548064

[B39] Lotte F. Congedo M. Lécuyer A. Lamarche F. Arnaldi B. (2007). A review of classification algorithms for EEG-based brain-computer interfaces. J. Neural Eng. 4, R1–R13. 10.1088/1741-2560/4/2/R01 17409472

[B40] Madhavan P. Wiegmann D. A. (2007). Similarities and differences between human–human and human–automation trust: an integrative review. Theoretic. Issues Ergon. Sci. 8, 277–301. 10.1080/14639220500337708

[B41] Madhavan P. Wiegmann D. A. Lacson F. C. (2006). Automation failures on tasks easily performed by operators undermine trust in automated aids. Hum. Fact. 48, 241–256. 10.1518/001872006777724408 16884046

[B42] Matoff-Stepp S. Applebaum B. Pooler J. Kavanagh E. (2014). Women as health care decision-makers: implications for health care coverage in the United States. J. Health Care Poor Underserved 25, 1507–1513. 10.1353/hpu.2014.0154 25418222

[B43] Mehta R. K. Parasuraman R. (2013). Neuroergonomics: a review of applications to physical and cognitive work. Front. Hum. Neurosci. 7:889. 10.3389/fnhum.2013.00889 24391575 PMC3870317

[B44] Mouloua M. Hancock P. A. (2019). Human Performance in Automated and Autonomous Systems: Current Theory and Methods. Boca Raton, FL: CRC Press. Available online at: https://www.google.com/search?client=firefox-b-d&q=Boca+Raton&stick=H4sIAAAAAAAAAOPgE-LUz9U3ME7LK09S4gAxk03LjLS0spOt9POL0hPzMqsSSzLz81A4VhmpiSmFpYlFJalFxYtYuZzykxMVghJL8vN2sDICAMcQcgZTAAAA&sa=X&ved=2ahUKEwjMia7EvqvyAhXNW80KHe_eD3YQmxMoATAjegQIPRAD

[B45] Nass C. Moon Y. (2000). Machines and mindlessness: social responses to computers. J. Soc. Issue 56, 81–103. 10.1111/0022-4537.00153

[B46] Parasuraman R. de Visser E. Wiese E. Madhavan P. (2014). Human trust in other humans, automation, robots, and cognitive agents: neural correlates and design implications. Proc. Hum. Fact. Ergon. Soc. Ann. Meet. 58, 340–344. 10.1177/1541931214581070

[B47] Parasuraman R. Sheridan T. B. Wickens C. D. (2008). Situation awareness, mental workload, and trust in automation: viable, empirically supported cognitive engineering constructs. J. Cogn. Eng. Deci. Mak. 2, 140–160. 10.1518/155534308X284417

[B48] Perrotta G. (2019). Anxiety disorders: definitions, contexts, neural correlates and strategic therapy. J. Neur. Neurosci. 6:15.

[B49] Pushparaj K. Ayeni A. J. Ky G. Alam S. Vijayaragavan V. Gulyás B.. (2019). A Quantum-Inspired Model for Human-Automation Trust in Air Traffic Control derived from Functional Magnetic Resonance Imaging. Available online at: https://www.semanticscholar.org/paper/A-Quantum-Inspired-Model-for-Human-Automation-Trust-Pushparaj-Ayeni/3ae85a2c776d0e8c145e39f7bd5886afd5227ebc

[B50] Roscoe A. H. (1992). Assessing pilot workload. why measure heart rate, HRV and respiration? Biol. Psychol. 34, 259–287. 1467396 10.1016/0301-0511(92)90018-p

[B51] Sanders N. Choo S. Kim N. Nam C. S. Fitts E. P. (2019). Neural correlates of trust during an automated system monitoring task: preliminary results of an effective connectivity study. Proc. Hum. Fact. Ergon. Soc. Ann. Meet. 63, 83–87. 10.1177/1071181319631409

[B52] Schaefer K. E. Billings D. R. Szalma J. L. Adams J. K. Sanders T. L. Chen J. Y.. (2014). A meta-analysis of factors influencing the development of trust in automation: implications for human-robot interaction: defense technical information center. Res. Artic. 58:228. 10.1177/0018720816634228 27005902

[B53] Strait M. Briggs P. Scheutz M. (2015). Gender, more so than age, modulates positive perceptions of language-based human-robot interactions. Available online at: https://www.semanticscholar.org/paper/Gender-%2C-more-so-than-Age-%2C-Modulates-Positive-of-Strait-Briggs/7e22cdd5af40239dd3f49147db7c67e342ab3b57

[B54] Syrdal D. S. Lee Koay K. Walters M. L. Dautenhahn K. (2007). A personalized robot companion? The role of individual differences on spatial preferences in HRI scenarios. RO-MAN 2007 16th IEEE Int. Sympo. Robot Hum. Interact. Commun. 1143–1148. 10.1109/ROMAN.2007.4415252

[B55] Trösterer S. Meschtscherjakov A. Mirnig A. G. Lupp A. Gärtner M. McGee F.. (2017). What we can learn from pilots for handovers and (de)skilling in semi-autonomous driving: an interview study. Proc. 9th Int. Conf. Autom. User Interfaces Interact. Vehicul. Appl. 173–182. 10.1145/3122986.3123020

[B56] Wang M. Hussein A. Rojas R. F. Shafi K. Abbass H. A. (2018). EEG-based neural correlates of trust in human-autonomy interaction. 2018 IEEE Sympo. Series Comput. Intell. 47, 350–357. 10.1109/SSCI.2018.8628649

[B57] Wickens C. D. Dixon S. R. (2007). The benefits of imperfect diagnostic automation: a synthesis of the literature. Theoretic. Issues Ergon. Sci. 8, 201–212. 10.1080/14639220500370105

[B58] Yanagisawa K. Masui K. Furutani K. Nomura M. Ura M. Yoshida H. (2011). Does higher general trust serve as a psychosocial buffer against social pain? an NIRS study of social exclusion. Soc. Neurosci. 6, 190–197. 10.1080/17470919.2010.506139 20706962

